# A genome-wide association analysis for body weight at 35 days measured on 137,343 broiler chickens

**DOI:** 10.1186/s12711-021-00663-w

**Published:** 2021-09-08

**Authors:** Christos Dadousis, Adriana Somavilla, Joanna J. Ilska, Martin Johnsson, Lorena Batista, Richard J. Mellanby, Denis Headon, Paolo Gottardo, Andrew Whalen, David Wilson, Ian C. Dunn, Gregor Gorjanc, Andreas Kranis, John M. Hickey

**Affiliations:** 1 The Roslin Institute, University of Edinburgh, Midlothian, UK; 2 Department of Animal Breeding and Genetics, Swedish University of Agricultural Sciences, Uppsala, Sweden; 3 Italian Brown Breeders Association, Loc. Ferlina 204, 37012 Bussolengo, Italy; 4 Aviagen Ltd, Midlothian, UK

## Abstract

**Background:**

Body weight (BW) is an economically important trait in the broiler (meat-type chickens) industry. Under the assumption of polygenicity, a “large” number of genes with “small” effects is expected to control BW. To detect such effects, a large sample size is required in genome-wide association studies (GWAS). Our objective was to conduct a GWAS for BW measured at 35 days of age with a large sample size.

**Methods:**

The GWAS included 137,343 broilers spanning 15 pedigree generations and 392,295 imputed single nucleotide polymorphisms (SNPs). A false discovery rate of 1% was adopted to account for multiple testing when declaring significant SNPs. A Bayesian ridge regression model was implemented, using AlphaBayes, to estimate the contribution to the total genetic variance of each region harbouring significant SNPs (1 Mb up/downstream) and the combined regions harbouring non-significant SNPs.

**Results:**

GWAS revealed 25 genomic regions harbouring 96 significant SNPs on 13 *Gallus gallus* autosomes (GGA1 to 4, 8, 10 to 15, 19 and 27), with the strongest associations on GGA4 at 65.67–66.31 Mb (Galgal4 assembly). The association of these regions points to several strong candidate genes including: (i) growth factors (GGA1, 4, 8, 13 and 14); (ii) leptin receptor overlapping transcript (*LEPROT*)/leptin receptor (*LEPR*) locus (GGA8), and the *STAT3*/*STAT5B* locus (GGA27), in connection with the JAK/STAT signalling pathway; (iii) T-box gene (*TBX3/TBX5*) on GGA15 and *CHST11* (GGA1), which are both related to heart/skeleton development); and (iv) *PLAG1* (GGA2). Combined together, these 25 genomic regions explained ~ 30% of the total genetic variance. The region harbouring significant SNPs that explained the largest portion of the total genetic variance (4.37%) was on GGA4 (~ 65.67–66.31 Mb).

**Conclusions:**

To the best of our knowledge, this is the largest GWAS that has been conducted for BW in chicken to date. In spite of the identified regions, which showed a strong association with BW, the high proportion of genetic variance attributed to regions harbouring non-significant SNPs supports the hypothesis that the genetic architecture of BW35 is polygenic and complex. Our results also suggest that a large sample size will be required for future GWAS of BW35.

**Supplementary Information:**

The online version contains supplementary material available at 10.1186/s12711-021-00663-w.

## Background

Poultry meat represents a major component of human nutrition [1]. At the beginning of 2020, the production and consumption of poultry meat surpassed those of pork and it is expected that in the next decade, poultry meat will account for nearly half of the additional produced meat [1, 2]. Body weight (BW) is one of the most economically important traits in the broiler industry. Traditional broiler breeding programs have achieved an increase in meat production efficiency of ~ 3.3% per year and selection on body weight has contributed to this result [3]. However, in spite of the importance of this trait, relatively little is known about the genetic variants that underlie the variance observed in body weight.

Knowledge of the genetic variants that underlie the variance observed in traits can amplify the breeding efficiency. For example, accuracy of genomic prediction can be increased by using markers that are strongly linked to the causative loci in genomic prediction models. Furthermore, information on the genetic architecture that underlies growth in meat type poultry will help to unravel the genes and pathways that are involved and enhance our understanding on such complex developmental processes.

Quantitative trait loci (QTL) mapping and genome-wide association studies (GWAS) have been used to improve BW in chicken [4–9]. However, although significant associations have been detected, their practical value to breeding programs is limited. Typically, the associations were not finely mapped and encompassed broad chromosome regions. Furthermore, the populations used were often F_2_ or advanced inter-crosses between lines of chicken that have been selected for egg laying, which are relatively slow growing, and fast growing broiler lines [4, 10, 11]. Relatively few publications on GWAS of body weight are available for commercially relevant lines [9, 12, 13].

Moreover, a large part of the genetic variance might be due to rare variants, or variants that are highly correlated/linked with other variants [14]. If this is the case, then a GWAS with a large sample size is required to detect those variants [15–18]. There is theoretical [19] and empirical evidence [16, 20, 21] that the power of GWAS increases as the size of the dataset increases. For example, a series of studies with datasets of increasing size for human height discovered 180 significant associations with a dataset of 183,727 individuals [15], 697 significant associations with a dataset of 253,288 individuals [16], and recently, another 83 new significant associations not previously detected were identified with a dataset of 711,428 individuals [21]. Analogous results are reported for studies on type 2 diabetes [22] and Crohn’s disease [20] in humans. If BW is a complex polygenic trait [23, 24], a large number of small-effect variants might regulate its expression. Hence, a large GWAS (in terms of sample size and number of markers analysed) is required to discover such variants.

The routine use of genomic selection in broiler breeding makes large GWAS possible. As part of the routine implementation of genomic selection over the past decade, Aviagen has accumulated both single nucleotide polymorphisms (SNP) array genotype and phenotype data for BW on 157,674 individuals from one of its lines.

Our objective was to conduct a GWAS, with a large sample size, for BW measured at 35 days of age (BW35) in broilers, which is a typical age at which broilers are slaughtered for meat production. After editing routinely collected data from a commercial broiler line, we analysed a dataset consisting of 137,343 broilers with phenotypes and 595,299 imputed SNPs.

## Methods

### Data

In total, we used 157,674 broilers spread across 15 generations of a pedigree for which BW35 and SNP array genotype data were collected as part of the routine commercial broiler breeding program (Aviagen Ltd, Newbridge, UK). The line used in this study was a female line (maternal side). The birds were genotyped with SNP arrays of different densities: 600 k SNPs for 1690 birds, 50 k SNPs for 59,773 birds, 42 k SNPs for 1507 birds, 3 k SNPs for 72,221 birds and 384 SNPs for 2152 birds. The development of these arrays is described in detail in [25]. Of these 632,439 SNPs, 52,408 are proprietary to Aviagen. We included all the SNPs in the analysis but do not show the base pair positions of the proprietary significant SNPs in our results, which represent 18 of the 96 significant SNPs). To unify the data from the different arrays and reach the highest density of 600 k, we imputed the genotypes of all broilers’ to the 600 k Affymetrix Axiom chip with the AlphaImpute software v1.9 [26, 27]. Broilers with more than 10% missing SNP genotypes were excluded from the analysis. Quality control of the SNPs was carried out using the PLINK v1.07 software [28]; SNPs with a call rate higher than 0.95 and a minor allele frequency higher than 0.01, that showed no extreme deviation from the Hardy–Weinberg proportions (*P* < 0.000001), and that were located on the *Gallus gallus* (GGA) autosomes 1 to 28 (except GGA16) were retained. After quality control, 137,343 birds and 392,255 SNPs remained for the analysis. The Galgal4 assembly in the Ensembl Genome Browser (version 85) was used to map the SNP positions on the genome (www.ensembl.org).

### Statistical analysis

#### Pedigree genetic analysis

Variance component and heritability estimates were based on a pedigree-based model using ASReml.v3 [29]:

1 $$\mathbf{y}=\mathbf{X}\mathbf{b}+\mathbf{Z}\mathbf{a}+\mathbf{e},$$

where $\mathbf{y}$ is a vector of BW35 records; $\mathbf{b}$ is a vector of fixed non-genetic effects (sex, mating group (the average genetic level of the parents) with 325 levels, the pen effects and hatch week with 381 levels); $\mathbf{a}$ is a vector of random additive genetic effects; $\mathbf{e}$ is a vector of random residuals; $\mathbf{X}$ and $\mathbf{Z}$ are design matrices linking phenotypes to effects. The model assumptions were $\mathbf{a}\sim N(\mathbf{0},\mathbf{A}{\upsigma }_{\mathrm{a}}^{2})$ and $\mathbf{e}\sim N(\mathbf{0},\mathbf{I}{\upsigma }_{\mathrm{e}}^{2})$, where $\mathbf{A}$ is the pedigree relationship matrix, $\mathbf{I}$ is an identity matrix and ${\upsigma }_{\mathrm{a}}^{2}$ and ${\upsigma }_{\mathrm{e}}^{2}$ are the additive genetic and residual variances, respectively. Heritability was estimated as the ratio of ${\upsigma }_{\mathrm{a}}^{2}$ to the total phenotypic variance (${\upsigma }_{\mathrm{a}}^{2}+{\upsigma }_{\mathrm{e}}^{2}$).

#### Genome-wide association study

The GWAS was conducted by single SNP regression while simultaneously correcting for the background polygenic effect using the GEMMA software [30]:

2 $$\mathbf{y}=\mathbf{1}{\mu} +\mathbf{w}\mathbf{b}+\mathbf{g}+\mathbf{e},$$

where $\mathbf{y}$ is a vector of BW35 records pre-corrected for the non-genetic effects of sex, mating group, pen, and hatch; *μ* is intercept, $\mathbf{w}$ is a column vector of genotypes for the SNP of interest with the corresponding allele substitution effect $\mathbf{b}$; $\mathbf{g}$ is a vector of random additive genomic (polygenic) effects; and $\mathbf{e}$ is a vector of random residuals. The model assumptions were $\mathbf{g}\sim N(\mathbf{0},\mathbf{G}{\upsigma }_{\mathrm{g}}^{2})$ and $\mathbf{e}\sim N(\mathbf{0},\mathbf{I}{\upsigma }_{\mathrm{e}}^{2})$, where $\mathbf{G}$ is the genomic relationship matrix calculated following the first method of VanRaden [31] and ${\upsigma }_{\mathrm{g}}^{2}$ and ${\upsigma }_{\mathrm{e}}^{2}$ are, respectively, the additive genomic and residual variances. Matrix $\mathbf{G}$ was constructed and eigen decomposed using an in-house Python script. The eigenvalues and eigenvectors were subsequently used in GEMMA via flag -d and -u, respectively. A false discovery rate (FDR; Benjamini and Hochberg) of 1% was adopted to account for multiple testing when declaring significant SNPs [32]. Manhattan and quantile–quantile (Q–Q) plots of the GWAS results were drawn in R [33] with the *qqman* package [34]. Annotation of all the significant SNPs was performed with the variant effect predictor (https://www.ensembl.org/Tools/VEP) program using the Ensembl database and the Galgal4 assembly. Moreover, genes located 1 Mb up/downstream of the top SNP in each genomic region that contained significant SNPs were annotated using the BioMart tool of the Ensembl database and the Galgal4 assembly (http://www.ensembl.org/biomart/martview/).

### Genetic variance partitioning by genomic region

Based on the GWAS results, the genome was partitioned into different regions that harboured significant and non-significant SNPs. Regions that contained significant SNPs were defined by considering the region 1 Mb upstream and 1 Mb downstream from the SNP with the highest p-value in each region. Due to closely located GWAS signals on GGA13 (13a and 13b) and GGA14 (14a and 14b), the two regions on each of these chromosomes were merged. To reduce the computational cost, we used all the significant SNPs from the 600 k Affymetrix Axiom chip and among the non-significant SNPs only those that overlap between the 50 k and 600 k Affymetrix Axiom chips. To estimate the variance explained by each region, a Bayesian ridge regression model was implemented using AlphaBayes [35] and the same inputs as for the GWAS, but analysing all the SNPs simultaneously. Posterior samples for SNP effects for each region were obtained from 50,000 Markov-chain Monte Carlo (MCMC) iterations with a burn-in period of 10,000 iterations. For each region and each iteration, breeding values were calculated from SNP effects and SNP genotypes, the variance of these regional breeding values was calculated and divided by the variance of the breeding values for the whole genome to estimate the proportion of the (additive) genetic variance explained per genomic region, accounting for linkage-disequilibrium within and between regions [36].

## Results

### Descriptive statistics and pedigree genetic parameters

**Fig. 1:**
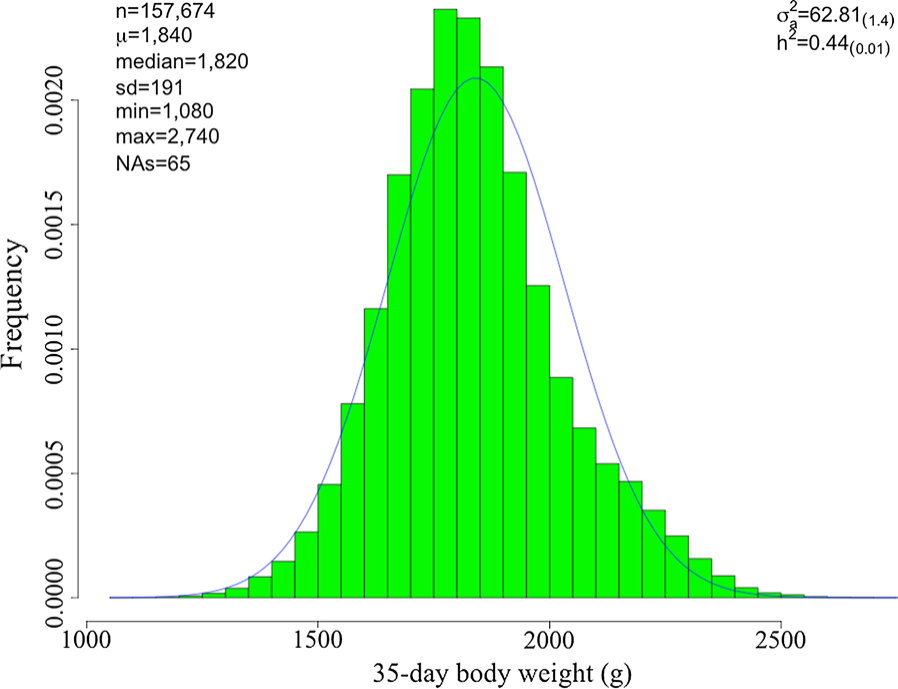
Descriptive statistics, additive genetic variance (${\sigma }_{a}^{2}$), and heritability (${h}^{2}$) estimates from the pedigree model

The summary statistics and variance components of the raw data are presented in Fig. 1. The average BW35 in the full dataset was 1840 g, and ranged from 1080 to 2740 g, while the estimated pedigree heritability was 0.44 (0.01).

### Genome-wide associations

**Fig. 2:**
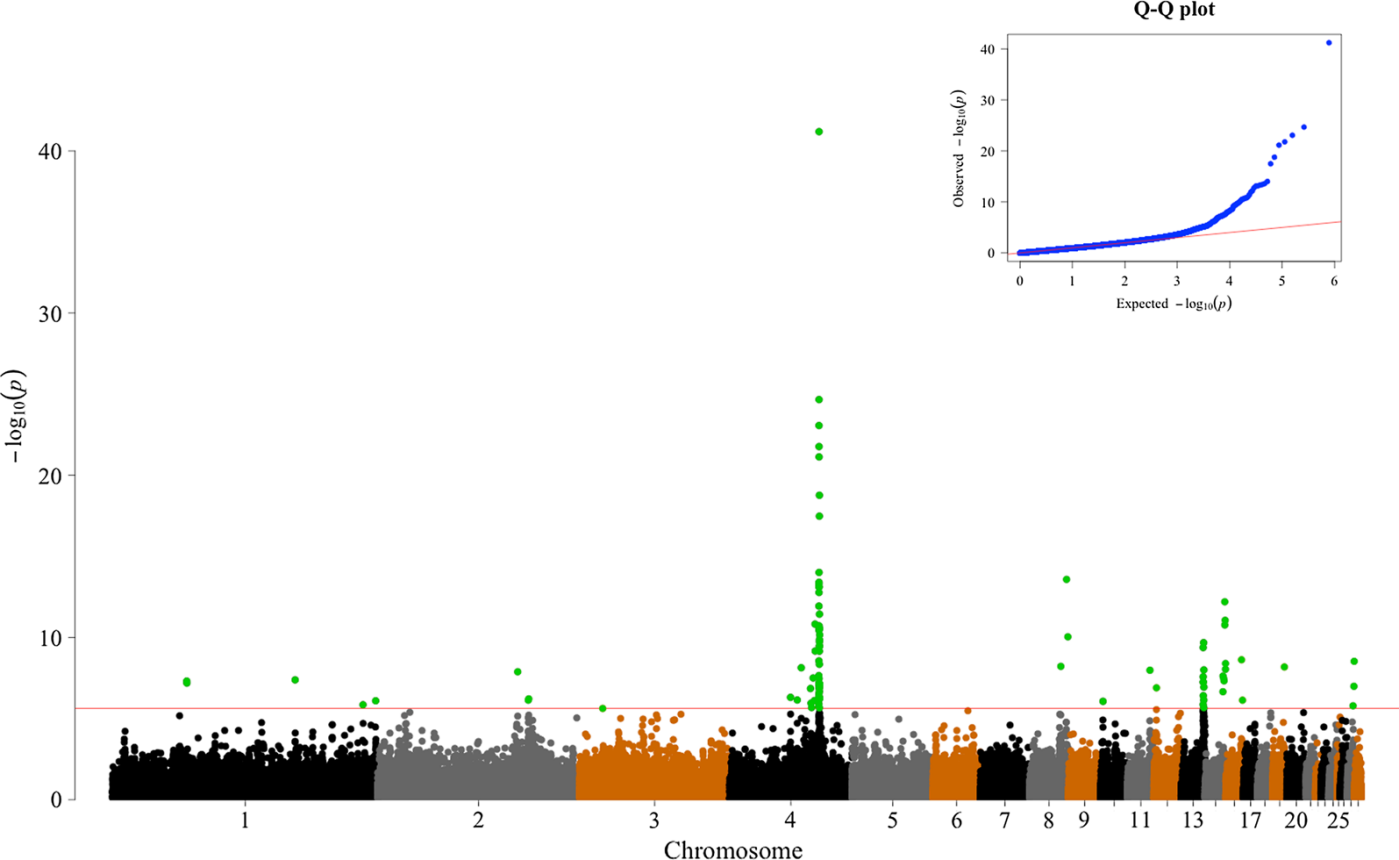
Manhattan plot of *P*-values for the genome-wide association study. A 1% false discovery rate was adopted to declare significance

**Fig. 3:**
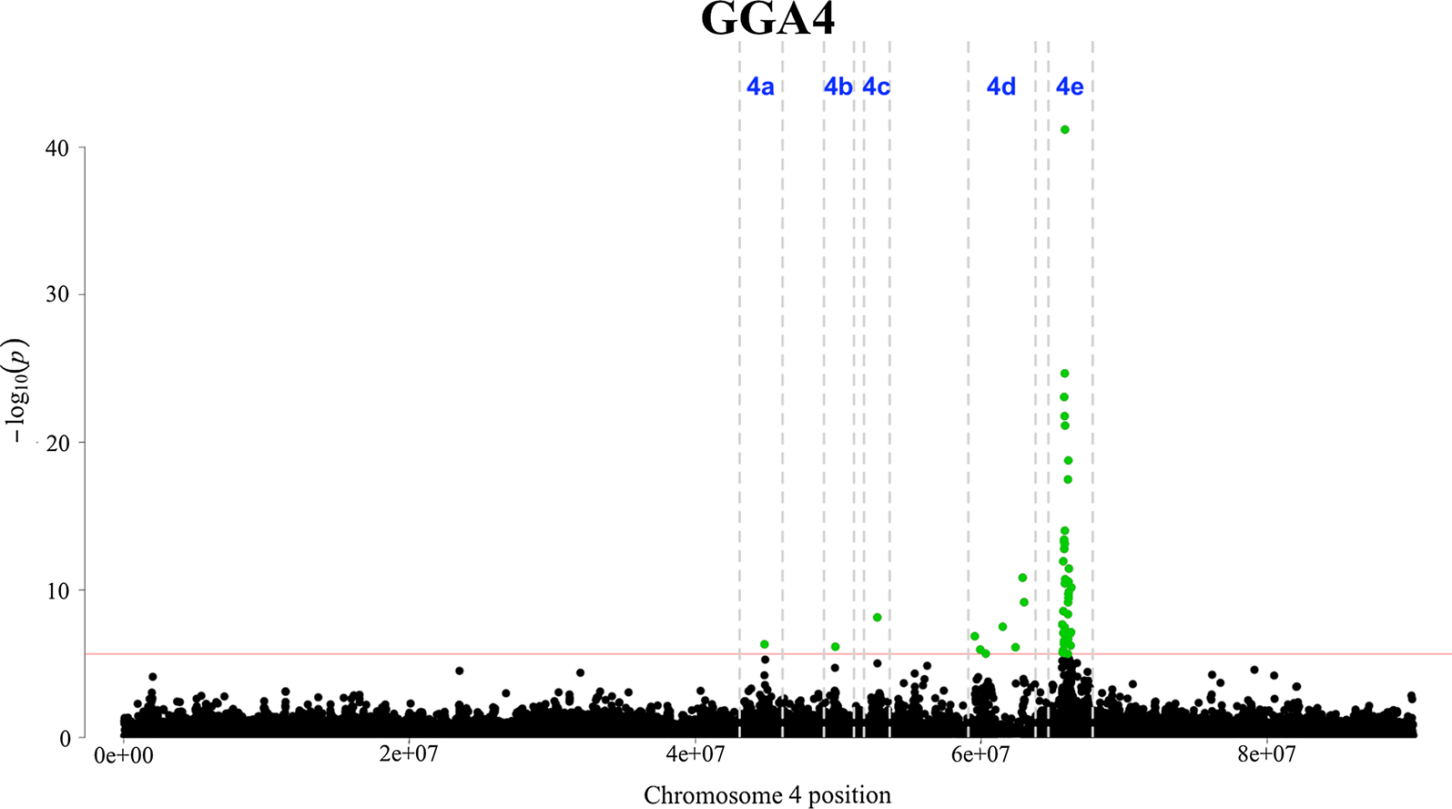
Manhattan plots of *P*-values for the genome-wide association study on *Gallus gallus* autosome 4. A 1% false discovery rate was adopted to declare significance

**Fig. 4:**
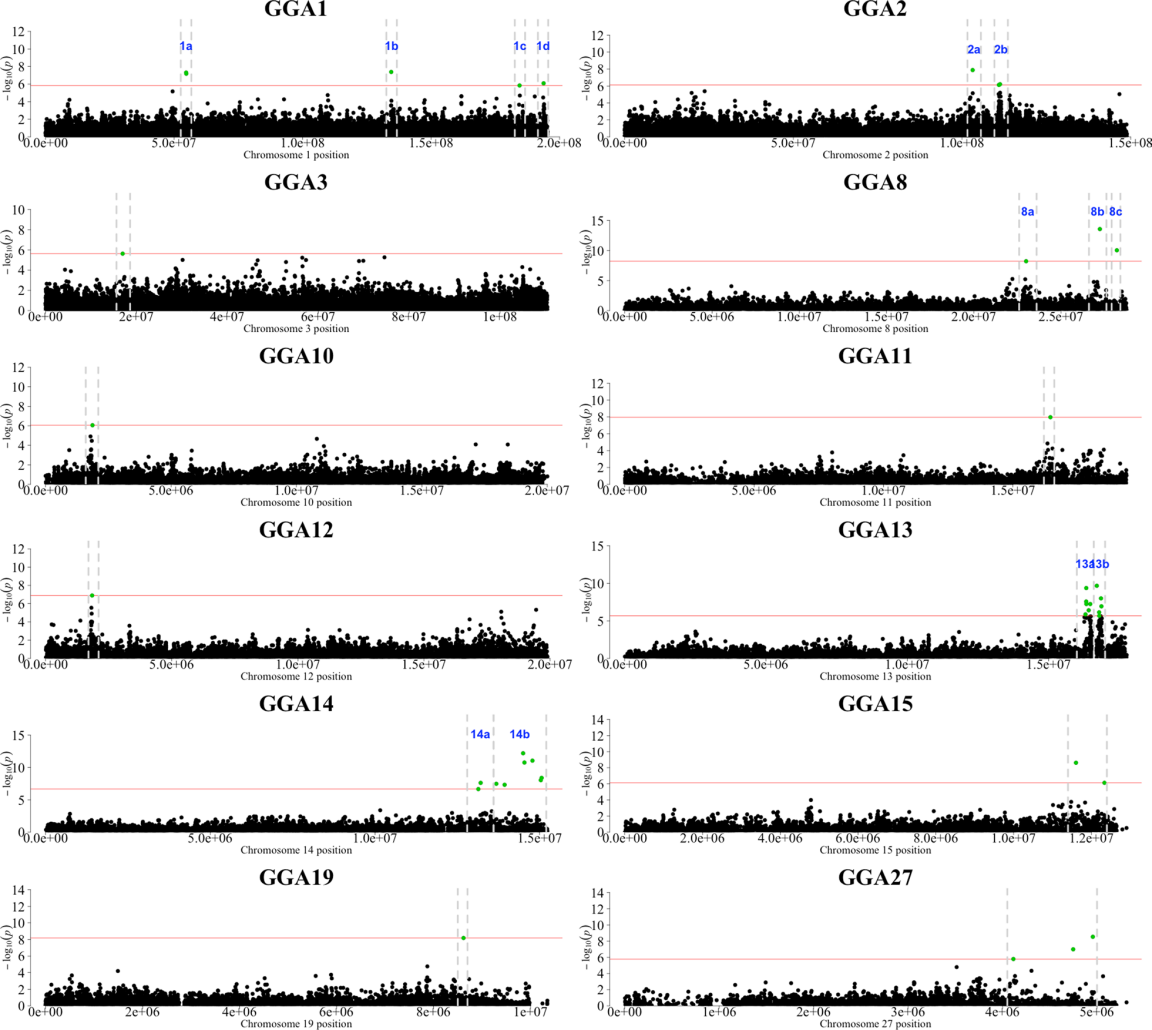
Manhattan plots of *P*-values for the genome-wide association studies on *Gallus gallus* autosomes (GGA) 1 to 3, 8, 10 to 15, 19 and 27. A 1% false discovery rate was adopted to declare significance

A summary of the results of the GWAS is in Table 1 and Figs. 2, 3 and 4. In total, 96 SNPs were significant at 1% FDR and distributed across 25 genomic regions on 13 chromosomes (GGA1 to 4, 8, 10 to 15, 19, and 27). Of these, 45 SNPs showed a very high significance and were located on GGA4 at ~ 65.86 Mb (Figs. 2 and 3). Details on these 96 significant SNPs including their chromosome and base pair location, their minor allele frequency and their effects and variances are in Additional file 1: Table S1. Of these 96 significant SNPs, 18 are proprietary to Aviagen and thus their base pair location is not shown in Additional file 1: Table S1.

**Table 1:** Summary of the genome-wide associations

GGA	Number of SNPs	Interval (Mb)	*P*-value (range)	*P*-value_FDR_ (range)	Top SNP location (bp)	Top SNP effect (SE)	Top SNP MAF
1a	2	54.68–54.76	4.90 × 10^–8^	3.49 × 10^–4^	54,681,614	− 0.64 (0.12)	0.18
1b	1	*/*	4.14 × 10^–7^	3.06 × 10^–4^	134,493,403	− 0.87 (0.16)	0.11
1c	1	*/*	1.42 × 10^–6^	6.18 × 10^–3^	184,458,596	0.76 (0.16)	0.11
1d	1	/	8.09 × 10^–7^	3.73 × 10^–3^	193,808,533	− 0.44 (0.09)	0.36
2a	1	/	1.31 × 10^–8^	1.12 × 10^–4^	103,154,441	0.44 (0.08)	0.41
2b	2	110.94–11.28	(7.40 × 10^–7^–6.17 × 10^–7^)	(3.51 × 10^–3^–3.06 × 10^–3^)	111,281,574	0.57 (0.11)	0.49
3	1	/	2.39 × 10^–6^	9.78 × 10^–3^	16,964,703	− 0.71 (0.15)	0.16
4a	1	/	4.96 × 10^–7^	2.56 × 10^–3^	44,839,695	0.67 (0.11)	0.34
4b	1	/	7.24 × 10^–7^	3.51 × 10^–3^	49,798,002	− 0.64 (0.13)	0.49
4c	1	/	7.41 × 10^–9^	6.92 × 10^–5^	52,734,745	− 0.67 (0.12)	0.31
4d	7	59.55–3.00	(2.12 × 10^–6^–1.51 × 10^–11^)	(8.94 × 10^–2^–3.11 × 10^–7^)	62,900,071	0.89 (0.13)	0.08
4e	45	65.67–66.31	(2.23 × 10^–6^–6.47 × 10^–42^)	(9.21 × 10^–3^–2.54 × 10^–36^)	^a^Proprietary	1.52 (0.11)	0.33
8a	1	/	6.09 × 10^–9^	5.97 × 10^–5^	22,999,302	− 1.05 (0.18)	0.08
8b	1	/	2.64 × 10^–14^	1.15 × 10^–9^	27,225,215	0.80 (0.11)	0.16
8c	1	/	9.16 × 10^–11^	1.38 × 10^–6^	28,197,569	− 1.35 (0.21)	0.22
10	1	/	8.37 × 10^–7^	3.98 × 10^–3^	1,870,252	− 0.42 (0.09)	0.39
11	1	/	1.06 × 10^–8^	9.20 × 10^–5^	16,452,035	0.41 (0.07)	0.43
12	1	/	1.28 × 10^–7^	7.48 × 10^–4^	1,843,370	0.52 (0.10)	0.32
13a	6	16.31–16.48	(1.37 × 10^–6^–4.22 × 10^–10^)	(6.03 × 10^–3^–5.17 × 10^–6^)	16,333,496	1.00 (0.16)	0.19
13b	5	16.71–16.87	(2.17 × 10^–6^–2.11 × 10^–10^)	(8.99 × 10^–3^–2.85 × 10^–6^)	16,706,244	0.79 (0.12)	0.46
14a	4	13.14–13.94	(2.23 × 10^–7^–2.40 × 10^–8^)	(1.23 × 10^–3^–1.96 × 10^–4^)	13,208,763	− 0.33 (0.06)	0.50
14b	5	14.50–15.06	(9.20 × 10^–9^–6.34 × 10^–13^)	(8.39 × 10^–5^–1.66 × 10^–8^)	14,496,748	− 0.53 (0.07)	0.30
15	2	11.61–12.34	(7.43 × 10^–7^–2.39 × 10^–9^)	(3.51 × 10^–3^–2.67 × 10^–5^)	12,338,040	0.32 (0.06)	0.26
19	1	/	6.64 × 10^–9^	6.35 × 10^–5^	8,618,001	− 0.83 (0.14)	0.25
27	3	4.11–4.96	(1.65 × 10^–6^–2.97 × 10^–9^)	(7.10 × 10^–3^–3.15 × 10^–5^)	4,955,965	0.40 (0.07)	0.31

^a^The top SNP in the region 4e was a proprietary SNP of Aviagen and therefore its base pair position is excluded

GGA = *Gallus gallus* chromosome; Number of SNPs = number of SNPs significantly associated to the trait; Interval = the chromosome region spanned by the significant SNPs (in base pairs); P-value (range) = the P-value of the highest significant SNP and the range of the P-values when multiple SNPs were significant; P-value_FDR_ (range) = false discovery rate P-value; Top SNP location (bp) = position of the most significant SNP on the chromosome; Effect (SE) = the allele substitution effect of the top SNP with the standard error in parenthesis; Top SNP MAF = minor allele frequency of the top SNP

On GGA1, four regions harboured significant SNPs, denoted 1a to 1d, at positions ~ 54.68 (1a), ~ 134.49 (1b), ~ 184.46 (1c) and ~ 193.81 Mb (1d). On GGA2, two regions harboured significant SNPs, denoted 2a and 2b, at ~ 103.15 (2a), ~ 111.28 Mb (2b) (Fig. 4). On GGA4, the most significantly associated SNP was located at ~ 65.86 Mb ($P=6.47\times {10}^{-42}$). The large region between ~ 65.67 and ~ 66.31 Mb (4e) contained 45 significant SNPs. Four more regions were significant on GGA4, at ~ 44.84 (4a), ~ 49.80 (4b), ~ 52.73 (4c) and ~ 59.55–63.00 Mb (4d) (Fig. 3). On GGA8, three significant SNPs were detected at ~ 23.00 (8a), ~ 27.23 (8b) and ~ 28.20 Mb (8c). At the tail of GGA13, two neighbouring regions were detected at ~ 16.33–16.48 Mb (13a) and ~ 16.71–16.87 Mb (13b). On GGA14, two closely located regions contained nine significant SNPs at ~ 13.14–13.94 Mb (14a) and ~ 14.50–15.06 Mb (14b). On GGA15, two SNPs were detected at ~ 11.61 (15a) and ~ 12.34 Mb (15b). On GGA27, three SNPs were significant at ~ 4.11–4.96 Mb. Moreover, five chromosomes had only one significant SNP: GGA3 (~ 17.00 Mb), GGA10 (~ 1.87 Mb), GGA11 (~ 16.45 Mb), GGA12 (~ 1.84 Mb) and GGA19 (~ 8.62 Mb) (Fig. 4).

### Partitioning of the genetic variance by genomic region

The proportion of the genetic variance over the total genetic variance for each genomic region that harboured significant SNPs is in Table 2. Combined together, the 25 genomic regions that harboured the 96 significant SNPs explained ~ 30% of the total genetic variance. Region 4e (GGA4 at ~ 65.67–66.31 Mb) that contained 45 significant SNPs explained the highest portion of the total genetic variance (4.37%). Regions with significant SNPs that explained the next largest amount of the total genetic variance were on GGA13 and GGA14, which each explained ~ 2.5%. All the other regions with significant SNPs each explained less than 2% of the total genetic variance. GGA4 explained more of the total genetic variance than any other chromosome. Taken together, the regions that harboured significant SNPs on GGA4 explained ~ 8.6% of the total genetic variance.

**Table 2:** Genetic variance explained by genome regions

GGA	V_A_	V_A_, %
1a	0.610^(0.30; 1.10)^	0.598^(0.38; 0.66)^
1b	0.402^(0.16; 1.11)^	0.395^(0.20; 1.40)^
1c	0.669^(0.16; 3.39)^	0.656^(0.20; 4.27)^
1d	1.395^(0.28; 5.64)^	1.368^(0.36; 7.10)^
2a	0.785^(0.37; 2.70)^	0.770^(0.47; 3.40)^
2b	0.841^(0.51; 1.26)^	0.824^(0.64; 1.58)^
3	0.973^(0.16; 5.55)^	0.954^(0.21; 6.99)^
4a	0.902^(0.36; 2.13)^	0.884^(0.46; 2.68)^
4b	1.674^(0.46; 6.91)^	1.642^(0.59; 8.70)^
4c	1.434^(0.29; 6.55)^	1.406^(0.37; 8.25)^
4d	0.283^(0.12; 0.70)^	0.278^(0.15; 0.88)^
4e	4.454^(2.96; 8.46)^	4.368^(3.73; 10.65)^
8a	0.865^(0.51; 1.47)^	0.849^(0.64; 1.85)^
8b	1.319^(0.73; 2.17)^	1.293^(0.92; 2.73)^
8c	0.911^(0.38; 1.94)^	0.893^(0.48; 2.44)^
10	1.299^(0.98; 1.77)^	1.274^(1.23; 2.23)^
11	1.473^(0.87; 2.60)^	1.444^(1.09; 3.27)^
12	1.315^(0.96; 1.74)^	1.289^(1.21; 2.20)^
13	2.588^(1.81; 3.87)^	2.538^(2.27; 4.87)^
14	2.513^(1.36; 4.72)^	2.465^(1.71; 5.95)^
15	0.937^(0.54; 1.76)^	0.919^(0.68; 2.21)^
19	1.579^(1.02; 2.53)^	1.549^(1.28; 3.19)^
27	1.143^(0.54; 2.56)^	1.121^(0.68; 3.22)^
Non-significant	71.603^(63.59; 95.44)^	70.222^(80.06; 120.15)^

GGA = *Gallus gallus* chromosome; V_A_: additive genetic variance by genomic region; V_A_, %: proportion of additive genetic variance explained by the genomic region; the parentheses denote the 95% high posterior density interval; non-significant SNPs were extracted from the 50 k Affymetrix Axiom chip; the two nearby located significant regions on GGA13 (13a and 13b) and GGA14 (14a and 14b) were merged on each chromosome

## Discussion

Previous studies have shown that the genomic architecture of BW is age- and population-dependent [8, 9, 37]. We focused on BW measured at 35 days of age, which is a typical age at which commercial broilers are slaughtered. In total, we found 96 significant SNPs with a 1% FDR that were located in 25 genomic regions across 13 chromosomes and explained ~ 30% of the genetic variance. We identified several candidate genes that might affect BW35 in broilers, and encode e.g. growth factors and the leptin receptor, and are involved in the JAK/STAT signalling pathway (Table 3). Furthermore, inspection of the Q–Q plot (Fig. 2) provided additional evidence of true associations, with an extreme departure observed at the tail of the distribution. We have divided the Discussion section into seven sub-sections: (i) summary of the QTL already known for BW35, (ii) growth factors, (iii) the leptin receptor overlapping transcript (LEPROT)/leptin receptor (*LEPR*) locus, (iv) the JAK/STAT signalling pathway, (v) the T-box genes (*TBX3*/*TBX5*), (vi) other candidate genes for BW35, and (vii) implications for breeding programs.

**Table 3:** List of genes associated with the significant SNPs

GGA	Location (Mb)	Gene name
1a	54.68–54.76	*CHST11*_*IN*_, *TXNRD1*_*IN*_, *IGF1*_*1Mb*_
1b	134,493,403	*C2orf49*_*3UR*_, *FHL2*_*DS*_, *TGFBRAP1*_*1Mb*_
1c	184,458,596	*MTMR2*_*IN*_
1d	193,808,533	*STIM1*_*IN*_, *DGAT2*_*1Mb*_
2a	103,154,441	*IMPACT*_*CL*_, *HRH4*_*CL*_
2b	110.94–111.28	*PLAG1*_*SYN*_, *IMPAD1*_*DS*_
3	16,964,703	*FBXO28*_*SYN*_
4a	44,839,695	*FGF5*_*1Mb*_, *BMP3*_*1Mb*_
4b	49,798,002	*SLC4A4*_*DS*_, *GC*_*1Mb*_
4c	52,734,745	*SPATA5*_*1Mb*_, *ADAD1*_*1Mb*_, *TRPC3*_*1Mb*_
4d	59.55–63.00	*METAP1*_*IN*_, *PPP3CA*_*IN*_, *ASAH1*_*3UR*_, F*GF20*_*1Mb*_
4e	65.67–66.31	*SPATA18*_*DS*_, *SGCB*_*DS*_*, DCUN1D4*_*IN*_, *CWH43*_*IN*_, *OCIAD1*_*IN*_, *SLAIN2*_*IN*_, *TEC*_*IN*_, *NFXL1*_*IN*_, *CORIN*_*IN*_
8a	22,999,302	*EPS15*_*IN*_
8b	27,225,215	*LEPROT*_*3UR*_, *JAK1*_*1Mb*_, *LEPR*_*1Mb*_
8c	28,197,569	*NEGR1*_*CL*_
10	1,870,252	*PPCDC*_*IN*_
11	16,452,035	*CRISPLD2*_*CL*_, *HNF4beta*_*CL*_
12	1,843,370	*MAPKAPK3*_*IN*_
13a	16.31–16.48	*KIF3A*_*US*_, *SEPT8*_*IN*_, *CCNI2*_*IN*_, *AFF4*_*IN*_
13b	16.71–16.87	*ARHGAP26*_*IN*_, *NR3C1*_*IN*_, *NDF1P1*_*1Mb*_, *FGF1*_*1Mb*_*,*
14a	13.14–13.94	*STUB1*_*US*_, *RHBDL1*_*IN*_, *JMJD8*_*DS*_, *SPSB3*_*IN*_, *NUBP2*_*US*_, *HN1L*_*DS*_, *IGFALS*_*1Mb*_
14b	14.50–15.06	*PDIA2*_*IN*_, *C16orf62*_*IN*_, *DNAH3*_*IN*_, *ZP2*_*MS*_
15	11.61–12.34	*MED13L*_*IN*_, *TBX5*_*IN*_
19	8,618,001	*SYNRG*_*IN*_, *TBX2*_*1Mb*_, *HNF1B*_*1Mb*_
27	4.11–4.96	*RPL19*_*US*_, *CACNB1*_*US*_, *DNAJC7*_*IN*_, *ATP6V0A1*_*IN*_, *STAT3*_*1Mb*_, *STATB5*_*1Mb*_

GGA = *Gallus gallus* autosome chromosome; Location = the chromosome region spanned by the significant SNPs (in base pairs); Gene name = names of associated genes; IN = intron variant; SYN = synonymous variant; DS = downstream gene variant; US = upstream gene variant; 3US = 3ʹ untranslated region; MS = missense variant; CL = closest gene (in the case of absence of genes within 5 kb from the top SNP); 1 Mb = important genes located within a 1-Mb range from the top SNP, as determined by the literature

### Summary of the QTL already known for BW35

A search on the animal QTL database (QTLdb; http://www.animalgenome.org/QTLdb) revealed a number of QTL for BW35 on GGA1 to 5, 7, 10–11, 15, 18, 20 and 27 (https://www.animalgenome.org/cgi-bin/QTLdb/GG/traitmap?trait_ID=2151). In particular, there is strong evidence in the literature for QTL associated with BW35 on GGA1 to 4.

Previous studies on F_2_ crosses between broiler and layer lines have identified QTL that are associated with a variety of carcass characteristics, such as BW measured at day 35, 41 and 63 [6, 7], and carcass and breast muscle yield [38, 39]. Nevertheless, such QTL studies detected only large chromosomal regions. The most significant chromosomal regions were mainly located on GGA1 to 4, but also GGA7 to 9, and GGA13, and on the Z chromosome [4, 5, 39].

Several GWAS have been conducted in broilers for a variety of carcass and growth traits, using commercial lines [9, 13], birds from experimental stations [7, 37, 40], or field data [41], and a variety of SNP array densities that ranged from ~ 44,000 [7–9] to 470,486 [41]. In spite of this, previous GWAS that scanned the entire genome, were still limited in terms of statistical power because the size of the samples was usually only a few hundreds of birds.

### Growth factor pathways

Growth factors, such as the transforming growth factor-β (TGF-β) and the insulin-like growth factor-1 (IGF1) are known to be key regulators of several traits related to body composition, growth, and development in chicken [42, 43]. Our analysis detected seven regions that include genes coding for growth factors, i.e. regions 1a and 1b, 4a, 4c, 4d, 13b, 14a (Table 3). More precisely, the *thioredoxin reductase 1* gene (*TXNRD1*; at ~ 54.74–54.77 Mb) is located in region 1a. The second most significant SNP detected in this region is located within *TXNRD1* (54,756,840 bp). Although TXNRD1 is not considered as a growth factor, studies on salivary adenoid cystic carcinoma [44] have found a synergistic action between TXNRD1 and TGF-β. The *insulin like growth factor 1* (*IGF1*; at ~ 55.43–55.48 Mb) gene is located within a 1-Mb region from *TXNRD1*. IGF1 has a major role in the body size of dogs [45, 46], with a single allele causing a small size. It also affects body size in mice [47] and height in humans [48, 49]. In broilers, increased IGF1 levels have been related with increased BW [43], growth of muscle [50], and IGF1 levels have been shown to differ between lines that are divergently selected for growth [51]. In a recent GWAS on a F2 chicken population, *TXNRD1* and *IGF1* have been associated with BW35 and BW41, respectively [52]. Moreover, the *IGFBP4* (*insulin like growth factor binding protein 4*) gene has been identified as a candidate gene for broiler BW in a study that analysed a subset of the population used in our work [13]. This gene is in close proximity (~ 0.5 Mb) to the signal we detected on GGA27. Another insulin-like growth factor modulating protein, namely that encoded by the *IGFALS* (*insulin-like growth factor binding protein, acid labile subunit*; ~ 13.2 Mb) gene, is located ~ 8 kb downstream of the region 14a. In addition, on GGA1, near the region 1b (~ 134.49 Mb), we identified the *TGFBRAP1* (*transforming growth factor beta receptor associated protein 1*; ~ 134.45–134.48 Mb) gene.

On GGA4, the region 4a (~ 44.84 Mb) contains two genes with growth factor activity: *FGF5* (*fibroblast growth factor 5*; ~ 44.74 Mb) and *BMP3* (*bone morphogenetic protein 3*; ~ 44.86 Mb). The *FGF20* gene, which is a key regulator of skin development in chicken [53], is also located on the same chromosome (region 4d; ~ 62.87 Mb), and the *FGF2* gene at ~ 53.00 Mb is close to the 4c region (~ 52.73 Mb). We detected another component of the growth factor pathway on GGA8, with the SNP in region 8a being located within the intronic region of the *EPS15* (*epidermal growth factor receptor pathway substrate 15*; ~ 22.96–23.0 Mb) gene. The region 13b is located between *NDF1P1* (*Nedd4 family interacting protein 1*; ~ 16.65–16.66 Mb) and another growth factor, *FGF1* (*fibroblast growth factor 1*; ~ 16.72–16.73 Mb).

### The leptin receptor overlapping transcript (LEPROT) and leptin receptor (LEPR)

Leptin (LEP) is a well-known hormone that is strongly related to appetite, through regulation of the brain satiety centres [54]. Its effect on reducing weight results from its interaction with another protein, the leptin receptor (LEPR) [55–57]. Several studies have investigated the association of the *LEP* and *LEPR* genes with obesity [58, 59], feed intake [60, 61], growth and fat traits [62, 63], and their cardio-metabolic implications [64, 65] in a variety of species. However, the results are controversial, especially in the chicken literature which currently reports evidence against the leptin system being involved in body weight control in birds [59, 66–68]. In our GWAS, a significant association in region 8b was located within the 3ʹ untranslated region of the *LEPROT* (*leptin receptor overlapping transcript*; ~ 27.22–27.23 Mb) gene. Moreover, the *leptin receptor* (*LEPR*; ~ 27.24–27.27 Mb) and the *JAK1* (*Janus kinase 1*; ~ 27.10–27.13 Mb) genes are located near this genomic region. It should be noted that *LEPROT* is not in any way homologous to the *leptin receptor* gene, but its expression has been associated with muscle development in turkey [69] and it is thought to regulate the expression of *cytokine receptor*, *growth hormone receptor* (*GHR*) and *LEPR* genes.

### The JAK/STAT signalling pathway

Although leptin activation of the JAK/STAT pathway in birds may not be important, as it is in other animals [70], this JAK/STAT pathway has a role in the mediation of many cytokine signals, such as those through GHR*,* and therefore for the growth of poultry. The JAK/STAT pathway is related to the generation of spermatogonial stem cells in chicken [71]. The association signal on GGA27 at ~ 4.96 Mb) (a gene dense region) was located near the *STAT3* (*signal transducer and activator of transcription 3*; ~ 4.90–4.91 Mb) gene, and more precisely is within an intron of the *ATP6V0A1* (*ATPase, H*+ *transporting, lysosomal V0 subunit a1*; ~ 4.93–4.96 Mb) gene (Table 3). The association between the *STAT3*/*STAT5B* locus and BW in chicken confirms the findings of [13]. We also detected several other genes related to reproduction. The association signal in region 4c (~ 52.73 Mb) is located near the *spermatogenesis associated 5* (*SPATA5*; ~ 52.76–52.98 Mb) gene. The *ADAD1* (*adenosine deaminase domain containing 1*; ~ 53.3 Mb) and *TRPC3* (*short transient receptor potential channel 3*; ~ 53.15 Mb) genes are located in the same region, and are involved in spermatid development and single fertilization. Interestingly, another spermatogenesis linked gene, *SPATA18* (~ 65.75–65.77 Mb), is located on GGA4 in the region with significant associations in our study (4e; ~ 65.67–66.31 Mb), that harbors two significant SNPs (at ~ 65.75 Mb) downstream of the gene (Table 3).

Apart from the effect of *LEPR* on the JAK/STAT pathway, Hou and Luo [64] have suggested a relationship between the leptin, JAK/STAT and mitogen-activated protein kinases (MAPK) signal pathways with an effect on cardiovascular diseases. Interestingly, the association on GGA12 was within an intron of the *MAPKAPK3* (*mitogen-activated protein kinase-activated protein kinase 3*; ~ 1.84–1.88 Mb) gene. The results of an experimental study in pigs that compared the expression level of *MAPKAPK3* in mini and large-type Diannan small-ear pigs, indicated that *MAPKAPK3* might have an important role in growth and development [72]. Interestingly, Tarsani et al. [13] suggested *LEMD2* (*LEM domain containing 2*), located on GGA26, as a strong candidate gene for BW and it is considered to have an important role during embryonic development in mice by regulating the MAPK signalling pathway [73].

### T-box genes (TBX5 and TBX3)

Several studies have implicated the effect of the T-box genes (*TBX4* and *TBX5*) in the development of the chicken limb, heart and embryo [74–77]. The significant SNP on GGA15 was included in the intron of *TBX5* (*T-box 5*; ~ 12.31–12.35 Mb). The *TBX3* gene is adjacent to *TBX5*. *TBX4* is located on GGA19 at ~ 7.59–7.61 Mb but the region that we detected on this chromosome is located further down at ~ 6.62 Mb, in the intron of the *SYNRG* (*synergin, gamma*) gene. In the same region on GGA19 (within 1 Mb) are found the *TBX2* (~ 7.63–7.64 Mb) and *HNF1B* (*HNF1 homeobox B*; ~ 8.64–8.66 Mb) genes. Interestingly, Tarsani and colleagues [13] have recently reported several candidate genes for BW35 in broilers, among which the *TBX21* gene and several members of the homeobox family (*HOXB1-9* and *HOXB13*), and Moreira et al. [52] have shown an association of *HOXB2,4,7,9* and *HOXB13* with BW35 in an F2 cross. However, it should be noted that all these genes are located on GGA27 at more than one Mb from our top SNP on that chromosome. In our analysis, another limb morphogenesis gene was detected in region 1a, with the SNP at 54,681,614 bp being within an intron of the *CHST11* (*carbohydrate (chondroitin 4) sulfotransferase 11*; ~ 54.54–54.74 Mb) gene (Table 3), which has been suggested to be involved in the elongation of limb buds and bone formation [78]. *CHST11* has also been recently reported as a candidate gene for BW35 [52].

### The zinc finger protein PLAG1

*PLAG1*, together with the *LCORL-NCAPG* locus, has been associated with body weight and height in a variety of species (human, cattle, horses, pigs and dogs) [79]. In cattle, a QTL for growth and development is located on bovine chromosome 14 (at ~ 25 Mb) and a cluster of four genes on the same chromosome, namely, *PLAG1*, *LYN* (*v-yes-1 Yamaguchi sarcoma viral related oncogene homolog*), *RPS20* (*ribosomal protein S20*), and *CHCHD7* (*coiled-coil-helix-coiled-coil-helix domain containing 7*) [80–84], is thought to be involved in this trait. Recently, a mutation in *PLAG1* has been reported to have a major contribution to stature in modern cattle [85]. More importantly, PLAG1 is also known to affect body weight and milk characteristics [86] and to regulate several growth factors, such as IGF2 [87, 88]. The large region 2b contains the *PLAG1*, *LYN,* and *RPS20* genes. Our top SNP on GGA2 was located 3.5 kb downstream of the *IMPAD1* (*inositol monophosphatase domain containing 1*) gene, but the second most significant SNP in the region was a synonymous variant in *PLAG1* (Table 3). Altogether, these findings mark *PLAG1* as a very good candidate for BW35 in broilers.

### Other candidate genes for BW35

#### On GGA4 (peak at ~ 65.86 Mb)

In line with previous studies [8, 89, 90], we found that GGA4 contains genes that play a role in body weight in chicken. Among these, the *CCKAR* (*cholecystokinin A receptor*) gene is located at ~ 72.8 Mb on GGA4. Decreased expression of this gene is associated with increased BW and growth in chicken and it has been suggested that modern chicken breeds might have been selected during the early domestication process for the high-growth haplotype [91]. Other studies have reported the *LCORL-NCAPG* locus, at ~ 74.0 Mb [12, 89], which explains part of the variance in stature in many species [92–96] and is associated with carcass traits in beef cattle [97]. However, the region identified in our study is located at ~ 65.86 Mb, which is quite far away (~ 7 Mb) from *CCKAR* and even further away from the *LCORL-NCAPG* locus. Whereas a long-range enhancer might be involved, a more conservative hypothesis indicates another gene. The top SNP in the region 4e detected here was ~ 400 bp upstream of the *CWH43* (*cell wall biogenesis 43 C-terminal homolog*) gene, which encodes the PGAP2-interacting protein. This SNP also had the highest positive effect, indicating an effect that increases BW35 (see Additional file 1: Table S1). Eight other SNPs in this region 4e are located in *CWH43*, among which four are in introns and two are synonymous variants. Forty-one SNPs in this region 4e were mapped to eight additional genes, some of these being located in their introns (Table 3), namely: *SPATA18*, *SGCB* (*sarcoglycan beta*), *DCUN1D4* (*DCN1, defective in cullin neddylation 1, domain containing 4*), *OCIAD1* (*OCIA domain containing 1*), *SLAIN2* (*SLAIN motif family member 2*), *TEC* (*tec protein tyrosine kinase*), *NFXL1* (*nuclear transcription factor, X-box binding like 1*), and *CORIN* (*corin, serine peptidase*). In the same region, five more genes are present, namely *FRYL* (*FRY like transcription coactivator*), *SLC10A4* (*solute carrier family 10 member 4*), *TXK* (*tyrosine kinase*), *NIPAL1* (*NIPA like domain containing 1*) and *CNGA1* (*cyclic nucleotide gated channel alpha 1*). Among all these genes, *SLAIN2* was recently reported as a strong candidate gene regulating BW in broilers [13]. Moreover, several members of the general solute carrier gene family have been associated with BW35 and BW41 [52].

Concerning the second region 4b on GGA4, we identified the *group-specific component* (*GC*) gene, which is located ~ 19 kb from the region (Table 3). GC (a vitamin D-binding protein) belongs to the general albumin family, involved in vitamin transportation, and vitamin D, lipids and lipoproteins metabolism and is expressed in all vertebrates [98]. In humans, it is one of the major determinants of the status in vitamin D, as assessed by measuring the circulating concentrations of 25 hydroxyvitamin D (25(OH)D) [99]. The role of vitamin D in the maintenance of skeletal health has been known for over a century but there is now growing evidence that vitamin D plays an important role also in the health of non-skeletal tissues. The linkage between *GC* and BW35 is consistent with recent studies that found that 25(OH)D supplementation increases breast meat yield in broilers [100]. This observation highlights the importance of understanding how key vitamin D metabolism pathways regulate physiological processes relevant to production in farm animals. In dairy cattle, the same gene was recently associated with complex traits such as mastitis and milk traits [101, 102].

#### On GGA1 to 3

Although the important role of GGA1 was previously reported in the literature, we detected only weak associations in our study, significant at 1% FDR. The top SNP in region 1b (~ 134.49) was located within the 3ʹ untranslated region of the *C2orf49* gene (~ 134.48–134.50 Mb), which is near the region containing the *C2orf40* gene. The SNP in region 1c was located within the *MTMR2* (*myotubularin related protein 2*; ~ 184.42–184.48 Mb) gene. In region 1d, several genes are mapped. The significant SNP located in region 1c was in an intron of the *STIM1* gene (*stromal interaction molecule 1*; ~ 193.79–193.83 Mb), which is close to *DGAT2* (*diacylglycerol O-acyltransferase homolog 2*; ~ 193.95–193.97 Mb), a gene that is related to fatty acid metabolism and associated with changes in carcass and meat quality characteristics in domestic pigeons [103].

On GGA2 two significant regions were detected, namely at 103.15 and 111.28 Mb. The closest genes in region 2a were *IMPACT* (*impact RWD domain protein*; ~ 103.03–103.05) and *HRH4* (*histamine receptor H4*; ~ 103.07) genes. On GGA3, the significant SNP was located within the *FBX028* (*F-box protein 28*; ~ 16.95–16.97 Mb) gene.

#### On chromosomes other than GGA1, 2, 3 and 4

We detected significant associations on nine other chromosomes (GGA8, 10, 11, 12, 13, 14, 15, 19 and 27). The association on GGA10 points to the *PPCDC* (*phosphopantothenoylcysteine decarboxylase*; ~ 1.86–1.88 Mb) gene, which is involved in the biosynthesis of coenzyme A. Coenzyme A is essential for energy production of the body. The association on GGA11 was located between the *CRISPLD2* (*cysteine-rich secretory protein LCCL domain containing 2*; ~ 16.37–16.40 Mb) and *HNF4beta* (*hepatic nuclear factor 4beta*; ~ 16.46–14.47 Mb) genes. On GGA13, two regions were identified and because of the “two-peak” pattern in the Manhattan plot (Fig. 3), they were considered as two different regions. The top SNP in region 13a is within the *SEPT8* gene (*septin 8*; ~ 16.31–16.40 Mb). On GGA14, in region 14b the significant SNP was located within the *PDIA2* gene (*protein disulfide-isomerase A2-like*; ~ 14.49–14.52 Mb). Finally, of particular interest are the signals on GGA27, on which apart from the *STAT3/STATB5* locus, we detected the *CACNB1* (~ 4.11 Mb; *calcium channel, voltage-dependent, beta 1 subunit*) gene, which has been reported as a strong candidate for BW35 in broilers [13] and for which skeletal muscle-specific isoforms are reported in humans [104].

### Implications for breeding programs

Combined together, the 25 genomic regions that contain 96 significant SNPs explained ~ 30% of the total genetic variance. This implies that the genetic architecture of BW35 is polygenic and complex, and therefore genomic prediction (using all available genomic data), rather than targeting specific genes via marker-assisted selection, will be more effective to improve BW35 in broilers. However, the region that contains significant SNPs and explains the largest proportion of the total genetic variance, is region 4e (~ 65.67–66.31 Mb), which explains 4.368% of the total genetic variance, and taken together the regions with significant SNPs on chromosome GGA4 explain ~ 8.6% (Table 2), which suggests that genomic prediction models that upweight regions of the genome known to harbour significant SNPs [105] may be effective. Moreover, information about the relevant genes identified in this paper could be included in the design of future SNP arrays.

## Conclusions

To the best of our knowledge, this is the largest GWAS that has been conducted for BW in chicken to date. Our analysis revealed 25 genomic regions that harbour 96 significant SNPs on 13 *Gallus gallus* autosomes, which combined together explain ~ 30% of the total genetic variance. Although the region on GGA4 at ~ 65.67–66.31 Mb explains 4.37% of the total genetic variance, the high proportion of genetic variance attributed to regions that harbour non-significant SNPs supports the hypothesis that the genetic architecture of BW35 is polygenic and complex. The significant SNPs and associated genes identified here could be used in future experimental designs targeting specific genes and biological pathways, and in the design of future SNP arrays as well as in statistical models of genomic prediction using prior biological knowledge of genome regions known to affect the traits of interest. Our results also illustrate the importance of a large sample size for future GWAS of BW35.

## Supplementary Information

**Additional file 1: Table S1.** List of all significant SNPs sorted by significance^1^. SNP = name of the single nucleotide polymorphism; GGA = *Gallus gallus* chromosome; location (bp) = position of the SNP on the chromosome in base pairs on the Galgal4 assembly; MAF = minor allele frequency; EFF = allele substitution effect; SE = standard error of the effect; P-value = *P*-values from the single SNP regression while simultaneously correcting for the background polygenic effect; *P*-value_FDR_ = false discovery rate *P*-value; LOG = the −log_10_ of the P-value; V_SNP_ = variance due to the SNP (calculated as 2pqb^2^, where p is the frequency of major allele, q = 1 − p is the frequency of the minor allele and b is the allele substitution effect); V_A_ = additive genetic variance; V_P_ = phenotypic variance; V_Asnp_ (%) = percentage of additive genetic variance explained by each SNP; V_Psnp_ (%) = percentage of phenotypic variance explained by each SNP. ^1^Of the 96 significant SNPs 18 are proprietary to Aviagen and therefore their base pair location has been excluded.
